# Isolation and Identification of Cancer Stem-Like Cells in Adenocarcinoma and Squamous Cell Carcinoma of the Lung: A Pilot Study

**DOI:** 10.3389/fonc.2019.01394

**Published:** 2019-12-18

**Authors:** Valentina Masciale, Giulia Grisendi, Federico Banchelli, Roberto D'Amico, Antonino Maiorana, Pamela Sighinolfi, Alessandro Stefani, Uliano Morandi, Massimo Dominici, Beatrice Aramini

**Affiliations:** ^1^Division of Thoracic Surgery, Department of Medical and Surgical Sciences for Children & Adults, University of Modena and Reggio Emilia, Modena, Italy; ^2^Division of Oncology, Department of Medical and Surgical Sciences for Children & Adults, University of Modena and Reggio Emilia, Modena, Italy; ^3^Rigenerand SRL, Modena, Italy; ^4^Department of Medical and Surgical Sciences for Children & Adults, Center of Medical Statistic, University of Modena and Reggio Emilia, Modena, Italy; ^5^Department of Medical and Surgical Sciences for Children & Adults, Institute of Pathology, University of Modena and Reggio Emilia, Modena, Italy

**Keywords:** cancer stem-like cells, non-small-cell lung cancer, lung adenocarcinoma, lung squamous cell carcinoma, CSC marker, aldehyde dehydrogenase

## Abstract

**Background:** Lung cancer stem cells (CSCs) share many characteristics with normal stem cells, such as self-renewal and multipotentiality. High expression of aldehyde dehydrogenase (ALDH) has been detected in many tumors, particularly in the CSC compartment, and it plays an important role in tumor proliferation, metastasis, and drug resistance. CD44 is commonly used as a cell surface marker of cancer stem-like cells in epithelial tumors. The aim of this study was to isolate and analyze cancer stem-like cells from surgically removed specimens to compare lung adenocarcinoma (ADENO) and squamous (SQUAMO) cell carcinoma.

**Methods:** The ALDEFLUOR assay was used to identify and sort ALDH^high^ and ALDH^low^ human lung cancer cells following tissue digestion. Fluorescence-activated cell sorting analysis for CD44 was performed with tumor cells. Quantitative real-time PCR was performed to assess the expression of SOX2 and NANOG as stemness markers. ALDH1A1 expression was additionally determined by immunohistochemistry. Anchorage-independent ALDH^high^ cell growth was also evaluated. ALDH^high^ ADENO and SQUAMO cells were cultured to analyze spheroid formation.

**Results:** All specimens contained 0.5–12.5% ALDH^high^ cells with 3.8–18.9% CD44-positive cells. SOX2 and NANOG relative expression in ALDH^high^ compared to ALDH^low^ cells in ADENO and SQUAMO was analyzed and compared between the histotypes. Immunohistochemistry confirmed the presence of ALDH1A1 in the sections. SOX2 and NANOG were expressed at higher levels in the ALDH^high^ subpopulation than in the ALDH^low^ subpopulation only in ADENO cells, and the opposite result was seen in SQUAMO cells. *In vitro* functional assays demonstrated that ALDH^high^ cells exhibited migration capacity with distinct behaviors between ALDH^high^ spheres in ADENO vs. SQUAMO samples.

**Conclusions:** Our results highlight the importance of a better characterization of cancer stem-like cells in ADENO and SQUAMO histotypes. This may suggest new differential approaches for prognostic and therapeutic purposes in patients with non-small-cell lung cancer.

## Background

Lung cancer is the most common cancer worldwide, accounting for 1.8 million new cases and 1.6 million deaths in 2012; the number of deaths worldwide is expected to grow to 3 million by 2035 (1, 2). Non-small-cell lung cancer (NSCLC) accounts for 85–90% of all lung cancers. The primary treatment is surgery for early stages (stages I and II) and chemotherapy, radiotherapy, and/or immunotherapy for advanced-stage disease (3–6). Chemotherapy drugs cannot differentiate between tumor cells and normal cells while functioning; the treatment-related adverse effects are noticeably strong and therefore feared by patients. It was not until the emergence of targeted therapy based on molecular typing that the survival period of patients with advanced NSCLC was improved to several years. Until 2013, immunotherapy was crowned as the first place scientific breakthrough (7). The efficacy of immunotherapy for those without targetable oncogene mutations was proven from second-line treatment (8–12) to first-line treatment (13, 14). Through long-term follow-up, immunotherapy has also shown that it has the greatest potential long-term clinical benefit (15, 16), even though the efficacy is not fully satisfactory (17–24). Indeed, similar to targeted therapy, patients may eventually develop resistance to immunotherapy (25, 26), and some may even suffer hyperprogression after immunotherapy (27, 28). The problem of resistance has not yet been studied; however, recent data suggest that cancer stem cells (CSCs) with characteristics of self-renewal may be resistant to these therapies (29). Understanding the role of CSCs in lung cancer may be very important and useful for identifying future targets. Indeed, the development of methods for the isolation and characterization of CSCs from primary tumors is a critical step in understanding the processes that mediate chemoresistance and for the development of therapeutic strategies to overcome this resistance, including promising immunotherapy approaches (29–31). To date, cancer cell lines have been the most frequently used tools to study lung CSCs (15, 16, 32). The identification of a specific marker for CSCs in the lung remains controversial (33, 34). Current studies provide increasing evidence for the existence of CSCs using several specific biomarkers (e.g., CD133, CD90, and CD44) translated from studies of human hematological malignancies (35–37) and solid tumors (38–47). In particular, aldehyde dehydrogenase (ALDH) activity is an important functional marker of normal and malignant stem/progenitor cells (47–51). In addition, CSCs possess high ALDH activity, especially for the predominant ALDH isozymes, ALDH1A1, and ALDH1A3. Cortes-Dericks et al. (51) showed that the flow cytometry-based ALDEFLUOR assay could be used to select ALDH^high^ and ALDH^low^ populations to discriminate the cancer stem-like cell population from non-cancer stem-like cells. An enrichment of CSCs in the ALDH^high^ population was also described in NSCLC patients and cell lines (52). In addition, several key regulators have been described as essential for the maintenance of a progenitor cell state under both normal and cancerous conditions (e.g., SOX2 and the homeobox protein NANOG) (53, 54). Following these investigations, the aim of the present study was to identify cancer stem-like cells in primary human lung cancer cells obtained from surgical specimens and to assess the differences and similarities between adenocarcinoma (ADENO) and squamous (SQUAMO) cell carcinoma using a combination of ALDH and CD44.

## Methods

The identification of cancer stem-like cells and the assessment of the differences and similarities between ADENO and SQUAMO were carried out by performing real-time PCR (RT-PCR), 3-(4,5-dimethylthiazol-2-yl)-2,5-diphenyltetrazolium bromide (MTS) assays, and sphere cultures.

### Collection of Tumor Specimens

This study was approved by the Regional Ethical Committee of Modena University Hospital and performed according to the guidelines of the Helsinki Convention. Upon signed informed consent, human lung cancer tissues were obtained from four consecutive patients with ADENO and four consecutive patients with SQUAMO who underwent major surgical lung resection between October 2017 and January 2018 at the Division of Thoracic Surgery of the University Hospital of Modena for stage I, II, or IIIA NSCLC (8th TNM) (Table 1). The collection of tumor tissues was carried out during surgery and was set according to the availability of the pathologists involved in our study. The excision of the tumor tissue was performed only from the primary lung nodule. The microscopic features of the cancer cells and immunohistochemistry were used to assess the histological diagnosis.

**Table 1:** Patients characteristics, cellular yield from each sample, viability, and aldehyde dehydrogenase (ALDH) expression determined by fluorescence-activated cell sorting (FACS).

		**Adenocarcinoma (*n* = 4)**	**Squamous cell carcinoma (*n* = 4)**	**All patients (*n* = 8)**
**PATIENTS CHARACTERISTICS**				
Age (years)	Mean ± SD	66.7 ± 9.4	73.7 ± 7.9	71 ± 8.5
Gender (male)	*n* (%)	3 (75.0%)	3 (75.0%)	6 (75.0%)
Smoker (yes)	*n* (%)	4 (100.0%)	4 (100.0%)	8 (100.0%)
Stage (8th TNM)				
IA3	*n* (%)	1 (25.0%)	1 (25.0%)	2 (25.0%)
IIA	*n* (%)	2 (50.0%)	0 (0.0%)	2 (25.0%)
IIB	*n* (%)	1 (25.0%)	1 (25.0%)	2 (25.0%)
IIIA	*n* (%)	0 (0.0%)	2 (50.0%)	2 (25.0%)
Neoadiuvant Chemotherapy	*n* (%)	1 (25.0%)	0 (0.0%)	1 (12.5%)
**SAMPLE CHARACTERISTICS**				
Weight (g)	Mean ± SD	1.0 ± 0.9	1.0 ± 0.6	1.0 ± 0.7
Cellular yield (million cells/g)	Mean ± SD	18.2 ± 8.6	20.4 ± 8.2	19.3 ± 7.9
**FACS ANALYSIS**				
7-AAD negative	Mean ± SD	94.3 ± 5.2%	90.5 ± 6.8%	92.4 ± 6.0%
ALDH^high^	Mean ± SD	3.7 ± 5.9%	4.2 ± 3.9%	4.0 ± 4.6%

*SD, standard deviation. 7-Amino-actinomycin D (7-AAD) negative cells are expressed as percentage of total number of sorted cells. ALDH^high^ are expressed as percentage of 7-AAD negative cells*.

### Dissociation of Primary Tissues

Freshly obtained tumor tissues (within 1–2 h after surgical removal) were washed in sterile Dulbecco's phosphate-buffered saline (PBS) (L1825-BC—Merck Millipore) and mechanically minced into small pieces (2–4 mm). Minced samples were digested using a tumor dissociation kit in a disposable gentle MACS™ C-Tube (Miltenyi) according to the manufacturer's instructions. Samples were digested for 60 min at 37°C in a gentle MACS Octo dissociator and filtered through 70-μm sterile cell strainers, centrifuged at 300 × *g* for 5 min, and resuspended in a mixture of Dulbecco's modified Eagle medium (DMEM) and Ham's F12 media (2:1) (Gibco) containing 50 IU/ml penicillin–streptomycin and 4 mM glutamine. Finally, viable cells were counted using an optic phase contrast microscope.

### ALDEFLUOR Assay

Single-cell suspensions of the primary tumor cells from the surgical tumor specimens were diluted in ALDEFLUOR assay buffer containing BODIPY-aminoacetaldehyde (STEMCELL Technologies, Vancouver, BC). The assay was performed according to the manufacturer's protocol. Briefly, at least 5 million tumor cells were resuspended in ALDEFLUOR buffer (5 μl/10^6^) and stained with ALDEFLUOR substrate. Immediately after, 5 × 10^5^ cells were transferred to a control tube containing 5 μl diethylaminobenzaldehyde, which is a specific inhibitor of ALDH. Both control and test samples were incubated for 45 min at 37°C protected from light. Following incubation, the cells were centrifuged at 300×*g* for 5 min. The cell pellet was resuspended in 1 ml ALDEFLUOR assay buffer. Cell morphology was evaluated using side scatter (SSC) and forward scatter (FSC). Dead cells were excluded using 7-amino-actinomycin D (7-AAD) staining. Cell sorting and ALDH analysis were performed using a FACSAria III instrument (Becton Dickinson, Franklin Lakes, NJ). The results were analyzed using fluorescence-activated cell sorting (FACS) Diva software (Becton Dickinson). The gating strategy included the ALDH^high^ gate, which was set at least one log apart from the ALDH^low^ gate. Sorted cells were promptly lysed for gene expression analysis.

### FACS Analyses

Primary tumor cell suspensions were stained with allophycocyanin-conjugated anti-CD45 (Becton Dickinson) and phycoerythrin-conjugated anti-CD44 (BioLegend, San Diego, CA). An isotype control sample for each condition was used to exclude the autofluorescence background. Dead cells were excluded using 7-AAD staining. The gate was set based on CD45-negative cells. Analyses were performed using a FACSAria III instrument (Becton Dickinson). Data were analyzed using the FACSDiva software.

### RNA Isolation and Real-Time PCR

Total cellular RNA was extracted from ALDH^high^ and ALDH^low^ cells using the RNeasy Mini Kit (Qiagen) according to the manufacturer's instructions. Total RNA (500 ng) was reverse transcribed using the RevertAid™ First-Strand Complementary DNA (cDNA) Synthesis Kit (Thermo Scientific). Following cDNA synthesis, RT-PCR was performed in triplicate for each sample using FAST SYBR™ Green detection chemistry (Applied Biosystems) on Step One instrument. Human SOX2, NANOG, and GAPDH were amplified using gene-specific primers (GAPDH: forward primer 5′-ACATCGCTCAGACACCATG-3′, reverse primer 5′TGTAGTTGAGGTCAATGAAGGG-3′; SOX2: forward primer 5′-GGAAACTTTTGTCGGAGACG-3′, reverse primer 5′-GCAGCGTGTACTTATCCTTC-3′; NANOG: forward primer 5′AGAAATACCTCAGCCTCCAG-3′, reverse primer 5′-CGTCACACCATTGCTATTCTT-3′). The cycling parameters consisted of denaturation at 95°C for 10 min; and 40 cycles of 15 s at 94°C, 30 s at 60°C, and 1 min at 72°C; followed by a continuous melting curve.

### Immunohistochemistry

Slides were deparaffinized with xylene, rehydrated in a graded alcohol series, and washed in PBS twice for 5 min each. The sections were heated in 10 mM sodium citrate buffer, pH 6.0, for 15 min in a 95°C water bath for antigen retrieval. PBS washes (5 min each) were performed until the buffer cooled down. Endogenous peroxidase activity was blocked via incubation in 3% H_2_O_2_ at room temperature for 10 min. Blocking serum was added in a dropwise manner at room temperature for 20 min to reduce the non-specific background. Samples were incubated with the anti-ALDH1A1 monoclonal antibody (ab-134188; 1:100 dilution; Abcam, Cambridge, MA, USA) overnight at 4°C. Sections were washed in PBS three times for 2 min and then incubated with a biotinylated secondary antibody (PK-4001; Vector Labs, USA) for 30 min at room temperature. The slides were subsequently incubated with ABC-HRP (PK-4001; Vector Labs, USA) for another 30 min, washed in PBS, and stained with 3,3-diaminobenzidine. Finally, the sections were counterstained with Mayer's hematoxylin, dehydrated, and mounted. Images were collected using a Zeiss Axioskop microscope with a Zeiss Axiocam ICc3 High-Resolution Microscope Camera. The scoring of the ALDH1A1 staining was performed by two independent investigators who were blinded to the patients' clinicopathological characteristics. Sections were scored independently. Immunoreactivity was scored using a semiquantitative method based on the ALDH positivity of the tumor cells as follows: 0 (<5% positive), 1 (5–25% positive), 2 (>25–50% positive), 3 (>50–75% positive), and 4 (>75% positive) (55).

### Cell Transformation Assay

Cell Biolabs CytoSelect™ 96-well cell transformation assay (cell recovery compatible, fluorometric) was used to analyze the anchorage-independent growth of ALDH^high^ cells, and the MCF-7 cell line was used as a positive control. ALDH^high^ cells were harvested and cultivated for a maximum of 48 h in appropriate serum-free medium, as described below. MCF-7 cells were cultivated in DMEM (Gibco) containing 50 IU/ml penicillin–streptomycin and 4 mM glutamine in the presence of 10% FBS (Euroclone). Cells were used at a concentration of 9,000 cells per well of the 96-well plate, and the growth kinetics on day 0 (T0) and day 8 (T8) were chosen to measure cell growth. This kit provided the soft agar material, solubilization solution, lysis buffer, and Cyquant^®^ GR Dye. The dye binds nucleic acids, and the relative fluorescence units (RFUs) were quantified to reveal a relative quantity of cells based on nucleic content.

### MTS Assay

The MTS Cell Proliferation Kit (Abcam) was used to measure the cell proliferation rate at six different time points after seeding (0, 1, 2, 7, 14, and 21 days). In a final volume of 200 μl of cell culture medium, 20 μl of MTS was added and incubated for 4 h at 37°C in standard culture conditions. After incubation, the optical density was measured at 490–500 nm by a Glomax Multi+ Detection System (Promega).

### Tumor Sphere-Forming Assay

ALDH^high^ and ALDH^low^ tumor spheres were dissociated into single-cell suspensions, and 50,000 cells from four different patients, two ADENO and two SQUAMO, were transferred to 24 ultralow attachment well plates. Cells were cultured in a mixture of serum-free DMEM and Ham's F12 media (2:1) (Gibco) containing 50 IU/ml penicillin–streptomycin and 4 mM glutamine supplemented with 5 μg/ml insulin, 10 ng/ml epidermal growth factor (EGF), 20 ng/ml basic fibroblast growth factor, 0.18 nM adenine, and 2 nM triiodotironin. The cells were cultured in 5% CO_2_ at 37°C for 2 weeks, and the media were replaced or supplemented with fresh growth factors twice a week. The entire well was digitally photographed using inverted phase-contrast microscopy (Zeiss Axioskop and Axiocam ICc3 color camera). All images were analyzed using the AxioVision software (Zeiss). The total number of spheres was counted, and sphere areas were manually measured at three different time points: 1, 2, and 3 weeks from seeding (56, 57).

### RT-PCR Data Analysis

We included four patients with ADENO and four patients with SQUAMO in the analysis, for a total of eight patients. This study uses a three-factor full factorial experimental design with replications, with factors such as (1) ALDH^high^ and ALDH^low^ cells; (2) SOX2, NANOG, and GAPDH genes; and (3) ADENO and SQUAMO histotypes. Replications are represented by triplicates. The analysis was performed using a linear mixed-model approach (58), which allows formal statistical hypothesis testing of relative gene expression. All cycle threshold values ≥36 were set as equal to 36. We assessed the relative messenger RNA (mRNA) expression of SOX2 and NANOG genes, normalized to the expression of the housekeeping gene, GAPDH, in ALDH^high^ cells compared to ALDH^low^ cells by means of a linear mixed regression model. The dependent variable was cycle threshold (Ct), whereas the independent variables were ALDH (high vs. low), gene (SOX2 and NANOG vs. GAPDH, which is the reference category), histotype (ADENO vs. SQUAMO), and pairwise and three-way interactions, all of which were analyzed as fixed-effect factors. The model also included a random intercept and a random ALDH–histotype interaction term that was specific for each patient to take into account correlations among cycle threshold values. The following parameters of interest were examined: (1) relative expression of SOX2 and NANOG in ALDH^high^ cells compared to ALDH^low^ cells in adenocarcinoma; (2) relative expression of SOX2 and NANOG in ALDH^high^ cells compared to ALDH^low^ cells in squamous cell carcinoma; and (3) differences between ADENO and SQUAMO in SOX2 and NANOG relative expression in ALDH^high^ cells compared to ALDH^low^ cells. The relative expression of SOX2 and NANOG in ALDH^high^ cells compared to ALDH^low^ cells, using GAPDH as the housekeeping gene, is reported as the fold change and the difference in cycle thresholds (equal to –log_2_ fold change) with 95% confidence intervals and *p*-values. Comparisons between ADENO and SQUAMO for SOX2 and NANOG relative expression in ALDH^high^ cells compared to ALDH^low^ cells are reported as *p*-values. All tests were two-sided *t*-tests using the Satterthwaite method for degrees of freedom.

### Statistical Analysis

Continuous variables were expressed as the mean ± standard deviation (SD) and the range, and categorical variables were expressed as absolute and percent frequencies. Statistical analyses of RT-PCR data were described in the previous paragraph. All statistical analyses were performed with R 3.4.3 software (The R Foundation for Statistical Computing, Wien) with *p* < 0.05 as the significance level.

### Sample Size

No formal sample size estimation was carried out in this pilot study. Therefore, the number of patients included in the analyses was only based on resource availability. The enrolled patients were those who underwent major surgical lung resection for stage I, II, or IIIA NSCLC in our division over a time period of 4 months. We included four consecutive patients with ADENO and four consecutive patients with SQUAMO to balance the two types of patients according to our full factorial experimental design.

### Genomics

Genomics data were routinely recorded in our Hospital for ADENO. DNA was extracted from formalin-fixed and paraffin-embedded blocks of each tumor sample. Extraction was performed with the QIAamp DNA Mini Kit (Qiagen, Hilden, Germany), and DNA was quantified with Xpose-NGS (Trinean NV, Gentbrugge, Belgium). Mutations were detected in genome-amplified DNA using the high-throughput genotyping platform Sequenom MassARRAY System (Sequenom, San Diego, CA, USA) and the Myriapod Colon Status Kit (Diatech Pharmacogenetics, Italy) following the manufacturer's protocol. This molecular array allows for the identification of the most important mutations of the *KRAS, NRAS, BRAF, EGFR, PIK3CA*, and *ERBB2* genes.

## Results

### Lung Cancer Tissues From Patients Yield Sufficient Numbers of Living Cells After Dissociation

All eight patients enrolled in the study (mean age, 71 ± 8.5 years old; range, 61–83; six male, two female, all smokers) underwent a lobectomy by lateral thoracotomy. Four patients were diagnosed with adenocarcinoma of the lung (mean age, 66.7 ± 9.4 years old; range, 61–79; three male, one female), and four patients (mean age, 73.7 ± 7.9 years old; range, 65–83; three male, one female) were diagnosed with squamous cell carcinoma (Table 1). Surgical tumor specimens (mean, 1.0 ± 0.7 g; range, 0.2–2.2) were obtained from each patient and used for the experiments, with similar specimen weights in the ADENO and SQUAMO groups (Table 1). A procedure combining mechanical dissociation with enzymatic degradation of the extracellular matrix that maintained tissue structural integrity was used to obtain single-cell suspensions from the surgical tumor specimens. The average cellular yield was a mean of 19.3 ± 7.9 million cells per gram (range, 10.0–30.3), with similar cellular yields in ADENO and SQUAMO (Table 1). Good cell viability was further confirmed by FACS analysis.

### ALDH-Positive Stem-Like Cells Were Identified in Primary Lung Cancer Tissue

**Figure 1:**
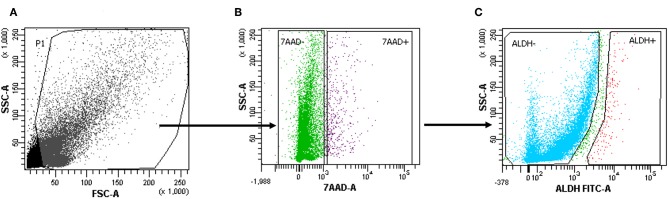
Cells with high aldehyde dehydrogenase (ALDH) activity in one patient sample (1.2%). **(A–C)** The gating strategy of a representative fluorescence-activated cell sorting (FACS) analysis of a primary tumor cell suspension in one patient. 7-Amino-actinomycin D (7-AAD) was used to assess ALDH^highorlow^ on the live population of cells.

Tumor tissue dissociation efficiently released cancer cells characterized by a heterogeneous morphology, as illustrated by widespread FSC and SSC values (Figure 1A). The mean viability of the samples was 92.4 ± 6.0% (range, 82.9–99.4%) based on 7-AAD staining (Table 1). These data further confirmed that the developed dissociation procedure was a non-toxic approach to isolating cancer cells from tumor tissues (Figure 1). The putative CSCs were physically separated from the bulk parental tumor cells and recovered by FACS according to the following gating strategy: Tumor cells were first identified based on their morphological parameters (FSC/SSC, Figure 1A, gate P1), and ALDH activity was measured in the 7-AAD-negative cell population only (Figure 1B). ALDH^low^ and ALDH^high^ cells were selected and sorted (Figure 1C). An ALDH^high^ subpopulation was identified in all samples (mean, 4.0 ± 4.6%; range, 0.5–12.5%, with two samples above 5%) of all viable lung cancer cells (Table 1), which indicates that it was possible to preserve lung cancer cells using a rapid dissociation protocol that allowed the identification of putative CSCs.

### Primary Lung Tumor Cells Express CD44

**Figure 2:**
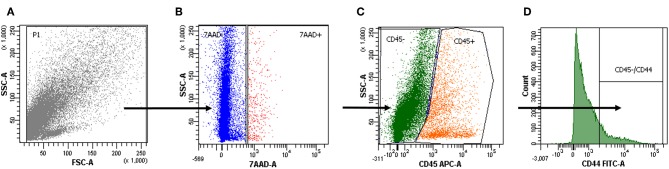
Cell positivity for CD44. **(A–D)** The gating strategy of a representative cytofluorimetric analysis of a primary tumor cell suspension. 7-Amino-actinomycin D (7-AAD) was used to exclude the CD45-positive cells in the live population to further analyze tumor cell positivity for CD44.

The surface marker CD44 was investigated as a possible marker for cancer stem-like cells. 7-ADD was used to identify viable cells, and CD45 staining was used to exclude CD45-positive cells (Figure 2) (59).

We found that ALDH^high^ and CD44-positive cells had comparable expression in our samples (4.0 ± 4.6 and 11.5 ± 7.7%, respectively), and there was a moderate positive correlation (Pearson correlation = 0.52).

### SOX2 and NANOG in ALDH^high/low^ Cells in Adenocarcinoma and Squamous Cell Carcinoma

**Figure 3:**
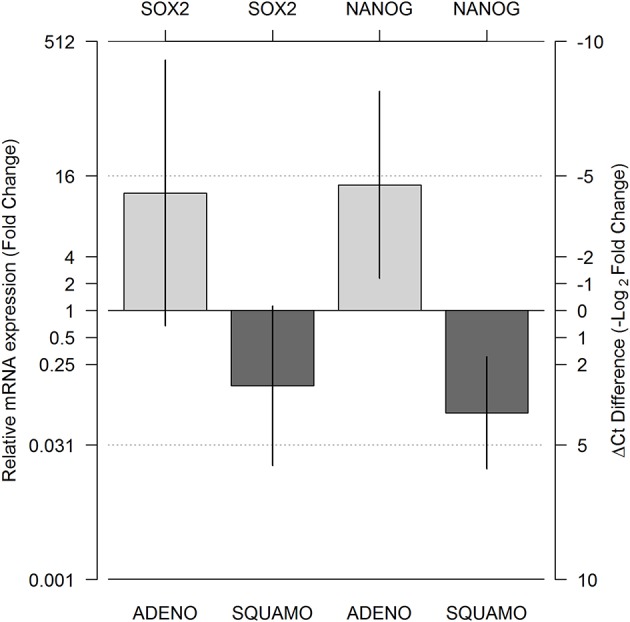
Relative messeger RNA (mRNA) expression of SOX2 and NANOG in ALDH^high^ compared to ALDH^low^ cell populations, using GAPDH as the reference gene. Expression was measured for the ALDH^low^ and ALDH^high^ cell populations in adenocarcinoma and squamous cell carcinoma histotypes using real-time PCR (RT-PCR), and relative expression comparing ALDH^high^ and ALDH^low^ was calculated by means of a linear mixed model. The two light gray bars represent ADENO, and the two dark gray bars represent SQUAMO. The error bars represent the 95% confidence intervals. ADENO, adenocarcinoma; SQUAMO, squamous cell carcinoma.

A total of 143 Ct values were available. Two patients (one ADENO and one SQUAMO) had SOX2 and NANOG Ct values in triplicate above 36 cycles in ALDH^low^ cells, and one ADENO patient had SOX2 and NANOG Ct values in triplicate above 36 cycles in ALDH^low^ cells and NANOG Ct values in triplicate above 36 cycles in ALDH^high^ cells. A total of 25 (17.5%) Ct values were above 36 and set equal to 36 for data analysis. The results from linear mixed-model analysis are reported in Table 2. The fold changes in ADENO were 20.72 (95% CI = 0.68; 635.58, *p* = 0.0755) and 25.49 (95% CI = 2.29; 283.44, *p* = 0.0147) for SOX2 and NANOG, respectively. The fold changes in SQUAMO were 0.14 (95% CI = 0.02; 1.13, *p* = 0.1022) and 0.07 (95% CI = 0.02; 0.31, *p* = 0.0073) for SOX2 and NANOG, respectively. These results are reported in Figure 3 by both the fold change and the cycle threshold difference scales. The differences in relative expression between ADENO and SQUAMO were statistically significant (*p* = 0.0101 and *p* = 0.0005 for SOX2 and NANOG, respectively).

**Table 2:** SOX2 and NANOG in ALDH^high/low^ cells.

	**ΔCt Difference (95% CI)**	**FC (95% CI)**	***p*-value**
**SOX2**			
Adenocarcinoma	−4.37 (−9.31; 0.57)	20.72 (0.68; 635.58)	0.0755
Squamous cell carcinoma	2.79 (−0.17; 5.76)	0.14 (0.02; 1.13)	0.1022
Difference			0.0101
**NANOG**			
Adenocarcinoma	−4.67 (−8.15; −1.20)	25.49 (2.29; 283.44)	0.0147
Squamous cell carcinoma	3.80 (1.71; 5.88)	0.07 (0.02; 0.31)	0.0073
Difference			0.0005

*95% CI, 95% confidence interval; FC, fold change. Relative gene expression of SOX2 and NANOG in ALDH^high^ cells compared to ALDH^low^ cells in adenocarcinoma and squamous cell carcinoma; results from linear mixed model analysis*.

### ALDH Positivity in Digested Samples Reflects Immunohistochemical Scoring in NSCLC

To further evaluate the ALDH expression pattern in the NSCLC samples, ALDH1A1 immunohistochemistry was scored as previously reported (55). Tissue sections were examined at 10 × magnification to characterize the overall staining pattern and at 20 × magnification for a more accurate evaluation of the cells to assign the appropriate values. As expected, normal bronchial epithelium and macrophages showed ALDH1A1 expression (49).

**Figure 4:**
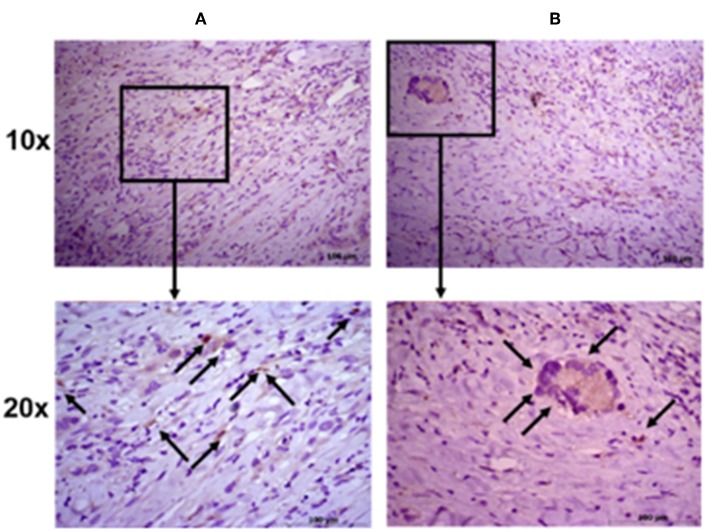
Immunohistochemical staining intensity of aldehyde dehydrogenase. Representative images of two patient samples in squamous cell carcinoma **(A)** and adenocarcinoma **(B)**. Images were taken at 10× (upper panels) and 20× (lower panels) magnification; black arrows indicate positive cells.

All eight patient samples showed a broadly similar intensity of ALDH1A1 staining in the cancerous fraction (i.e., all had scores of 0 with <5% positive tumor cells), which is consistent with the FACS data (Figure 4).

### Cell Transformation

**Figure 5:**
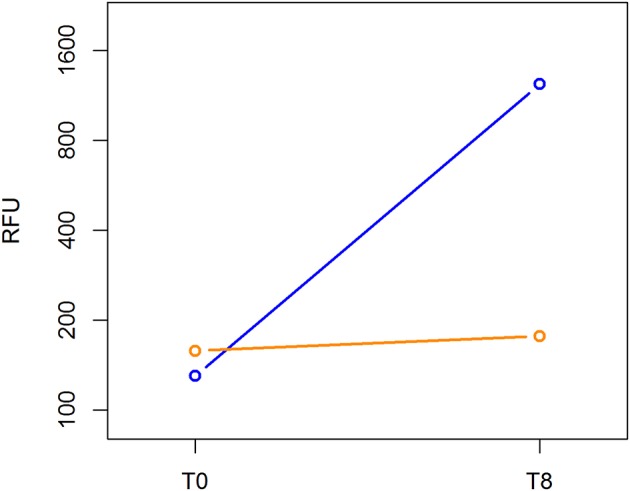
Cell transformation assay. The MCF-7 cell line and ALDH^high^ cells were compared for growth ability in a semisolid agar substrate. Two different time points were evaluated: 0 and 8 days. In the vertical axis, DNA content of each sample was measured using a fluorescent signal released by Cyquant GR Dye. Time points are represented in the horizontal axis.

The Cell Biolabs CytoSelect™ 96-well cell transformation assay did not involve subjective manual counting of colonies, but it used a fluorescent measurement of Cyquant^®^ GR Dye that bound to nucleic acids to quantify the number of cells based on nucleic assay content. We compared the cell proliferation at T0 and T8 between the MCF-7 breast cancer cell line and ALDH^high^ cells. The MCF-7 cell line grew from 130 to 1,233 RFU, and the ALDH^high^ cells rose mildly from 158 to 177 RFU (Figure 5).

### MTS Cell Proliferation Assay

**Figure 6:**
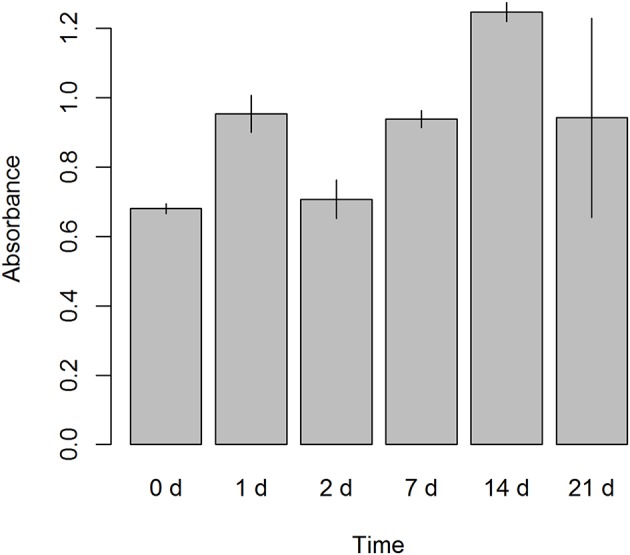
Cell proliferation assay. A colorimetric assay, 3-(4,5-dimethylthiazol-2-yl)-2,5-diphenyltetrazolium bromide (MTS) based, was performed on ALDH^high^ cells after seeding. Six different time points were evaluated (0, 1, 2, 7, 14, and 21 days). Absorbance was measured at 490 nm.

Cell proliferation was evaluated in ALDH^high^ cells from seeding (day 0) until the end of the culture (day 21). An MTS-based assay revealed that our culture protocol did not affect the proliferation of ALDH^high^ cells. There was a trend of growth that extended until day 21, without any notable drop in cell growth over time (Figure 6).

### General Characteristics of Tumor Spheres

ALDH^high^ cells of the ADENO and SQUAMO patients were maintained in low attachment cultures in the absence of serum for up to 3 weeks (Figure 7.1).

**Figure 7:**
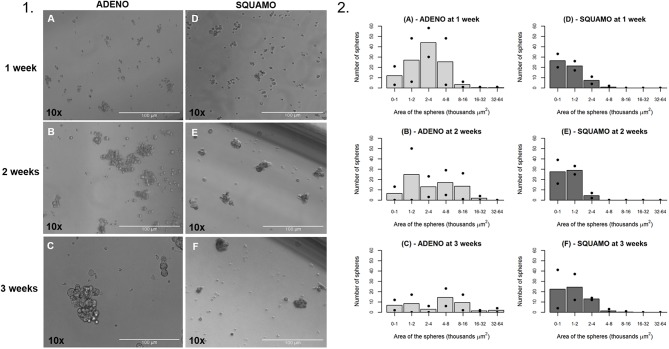
General characteristics over time of tumor spheres in adenocarcinoma and squamous cell carcinoma. **(1A–C)** ALDH^high^ spheres from ADENO at 1, 2, and 3 weeks. **(1D–F)** ALDH^high^ spheres from SQUAMO at 1, 2, and 3 weeks. **(2A–F)** The number of spheres and their areas were calculated for two adenocarcinoma patients and two squamous cell carcinoma patients 1, 2, and 3 weeks after seeding. Points represent values for individual patients, and bars represent average values. ADENO, adenocarcinoma; SQUAMO, squamous cell carcinoma.

**Figure 8:**
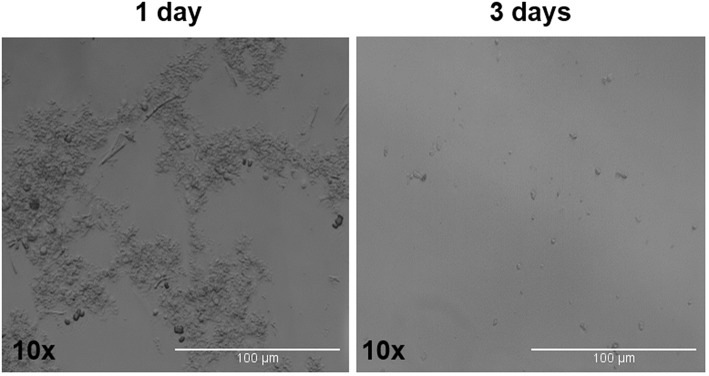
ALDH^low^ cells phase-contrast microscopy. ALDH^low^ cells in low-attachment serum-free culture showed difficult and slow growth that completely stopped at 3 days.

The tumor spheres that formed in each well were counted and measured for area in ADENO and SQUAMO patients at three different time points (1, 2, and 3 weeks), as shown in Figure 7.2. There was a tendency for ADENO to produce a higher number of spheres than SQUAMO, and the spheres produced by ADENO also exhibited greater area than SQUAMO. We observed that the spheres in ADENO had a tendency to grow in area and form larger spheres at 2 and 3 weeks, but there was no evidence of this in SQUAMO spheres, whose distribution did not significantly change over time. In contrast, ALDH^low^ cells of both histotypes died within 3 days, as shown in Figure 8.

### Genomics

Genomics data were recorded for four ADENO patients. Two of them had a KRAS mutation, one had an EGFR mutation and one patient was wild type for the analyzed mutations.

## Discussion

The CSC theory elucidates the origin of tumors, tumor development, metastasis, relapse, and drug resistance (60, 61). Therefore, the establishment of a reliable and efficient method for the isolation, manipulation, and characterization of CSCs is controversial, presumably due to the difficulty of identifying a specific marker. Thirty years ago, Carney and colleagues described a rare population of cells (<1.5%) in small and NSCLC samples that formed colonies in soft agar (62). When inoculated into athymic nude mice, these cells recapitulated the original lung cancer, which suggested that they had progenitor cell features (63). Over the last decade, several investigators isolated tumorigenic cell lines from lung cancers using different phenotypic cancer cell characteristics (48).

In the past, different methods have been used to identify CSCs, such as side population analysis, selection in culture, and cell sorting for a specific marker (64–67). Of all the markers explored, the CD133 marker has received the most attention (48). However, in our samples, the CD133 marker was not useful because of a lack of detection, as described previously (48). Consequently, we analyzed our population for CD44, which is a transmembrane receptor for hyaluronic acid that is a CSC marker of several stem cell-like properties (68). In addition, we used the ALDEFLUOR assay to isolate cancer stem-like cells, as previously described by Sullivan et al. (52). Interestingly, our results confirmed comparable ALDH^high^ and CD44 positive expression.

However, Sullivan et al. identified CSCs in a panel of 11 NSCLC tumor samples, 45 NSCLC lines, and 7 SCLC lines (52) that are used to study ALDH activity and sorted a subpopulation of NSCLC stem-like cells dependent on Notch signaling. Our study used the same method (52), but we focused on analyzing the differences and similarities between adenocarcinoma and squamous cell carcinoma cancer stem-like cells. Our hypothesis was supported by the fact that these populations are the most frequent histotypes in lung cancer patients and account for 50% of adenocarcinoma patients and 30% of squamous cell carcinoma.

We investigated the ALDH^high/low^ populations in both histotypes for the mRNA expression of SOX2 and NANOG, which are stemness-related genes in normal and cancer cells (60, 61).

The RT-PCR data from our patients revealed more SOX2 and NANOG expression in ALDH^high^ cells than in ALDH^low^ cells in adenocarcinoma. However, the opposite result was obtained for squamous cell carcinoma, in which lower SOX2 and NANOG expression was found in ALDH^high^ cells than in ALDH^low^ cells. Therefore, there was a concordant trend for SOX2 and NANOG relative mRNA expression, even though only the relative expression of NANOG reached statistical significance. There was a statistically significant difference between the relative mRNA expression of SOX2 and NANOG in adenocarcinoma compared to squamous cell carcinoma. The expression of these genes was discussed in previous attempts to find a connection between these stemness genes and the clinicopathological features of the tumor (68–70). Therefore, our study adds a new aspect by considering the existence of different cancer-stem-like cell populations for these two histotypes. However, our data suggest an enrichment of cells with stemness characteristics.

To further confirm CSC-like phenotypes, we analyzed the ability of cells to form tumor spheres in serum-free low-attachment cultures. Tumor sphere formation assays revealed a different pattern in sphere formation, dimension, and growth between adenocarcinoma and squamous cell carcinoma. The former had a tendency to produce a greater number of spheres and larger spheres than the latter. Moreover, we observed growth of adenocarcinoma spheres until the third week, but spheres from the squamous cell carcinoma did not increase in number or size. This result may be related to the distinct aggressiveness and clinicopathological characteristics of adenocarcinoma compared to squamous cell carcinoma.

Furthermore, genomics data highlighted the presence of genetic mutations in ADENO; however, the relationship of these mutations with the cancer stem-like cells in our study is unclear.

## Limitations of the Study

The main limitation of the study is the low number of patients included in the analyses (four ADENO and four SQUAMO). The selection of these few patients could have affected our results as well as the statistical power. Nevertheless, we have included consecutive patients—which prevents from selection bias—and we have observed statistically significant differences in gene expression between ADENO and SQUAMO, which partially counterbalances the issue regarding the low statistical power. Moreover, in this study, test samples were obtained by primary cell cultures derived from patients, which is more difficult to obtain than tumor cell lines. On the basis of these limitations, the results obtained in our pilot study should be confirmed by more extensive studies.

## Conclusion

Even with limited evidence due to the low number of patient samples, our study showed differences between adenocarcinoma and squamous cell carcinoma related to the analyzed stemness genes. ALDH^high^ cancer stem-like cells in adenocarcinoma showed stemness characteristics in gene expression and spheroid culture studies, but squamous cell carcinoma stemness characteristics were not completely clear because of the discrepancy between genes and cellular behavior.

To summarize, our results highlight the importance of a better characterization of cancer stem-like cells in ADENO and SQUAMO histotypes. This may suggest new differential approaches for prognostic and therapeutic purposes in patients with NSCLC.

## Data Availability Statement

The datasets generated for this study are available on request to the corresponding author.

## Ethics Statement

The studies involving human participants were reviewed and approved by Ethics committee at University Hospital of Modena, MODENA, Italy, on 17 March 2017, Prot. No. 914/C.E. The patients/participants provided their written informed consent to participate in this study.

## Author Contributions

The idea for the manuscript was conceived in September 2016 by BA and MD and was further developed by VM, GG, FB, RD'A, AM, and AS. AM and PS were involved in histopathological diagnosis. BA, VM, and FB wrote the first draft of the manuscript. BA and UM have been involved in surgery and tissue collection. VM and GG performed laboratory experiments, whereas FB and RD'A performed the statistical analysis. BA, VM, FB, MD, RD'A, AM, and UM reviewed the manuscript and were involved in its critical revision before submission. All authors read and approved the final manuscript.

### Conflict of Interest

The authors declare that the research was conducted in the absence of any commercial or financial relationships that could be construed as a potential conflict of interest.

## Abbreviations

NSCLC: non-small cell lung cancer
SCLC: small cell lung cancer
ALDH: aldehyde dehydrogenase
FACS: fluorescence-activated cell sorting
CSCs: cancer stem cells
SSC: side scatter
FSC: forward scattered
ECM: extracellular matrix
ADENO: adenocarcinoma
SQUAMO: squamous cell carcinoma.

## References

[1] Ferlay J Soerjomataram I Dikshit R Eser S Mathers C Rebelo M. Cancer incidence and mortality worldwide: sources, methods and major patterns in GLOBOCAN 2012. Int J Cancer. (2015) 136:E359–86. 10.1002/ijc.29210 25220842

[2] Didkowska J Wojciechowska U Manczuk M Łobaszewski J. Lung cancer epidemiology: contemporary and future challenges worldwide. Ann Transl Med. (2016) 4:150. 10.21037/atm.2016.03.11 27195268 PMC4860480

[3] Vansteenkiste J Crino L Dooms C Douillard JY Faivre-Finn C Lim E ESMO Consensus guidelines: early stage non-small cell lung cancer consensus on diagnosis, treatment and follow-up. Ann Oncol. (2014) 25:1462–74. 10.1093/annonc/mdu089 24562446

[4] Ettinger DS Wood DE Aisner DL Akerley W Bauman J Chirieac LR. Non-small cell lung cancer, version 5.2017, NCCN clinical practice guidelines in oncology. J Natl Compr Canc Netw. (2017) 15:504–35. 10.6004/jnccn.2017.0050 28404761

[5] Zappa C Mousa SA. Non-small cell lung cancer: current treatment and future advances. Transl Lung Cancer Res. (2016) 5:288–300. 10.21037/tlcr.2016.06.07 27413711 PMC4931124

[6] Snyder V Reed-Newman TC Arnold L Thomas SM Anant S. Cancer stem cell metabolism and potential therapeutic targets. Front Oncol. (2018) 8:203. 10.3389/fonc.2018.00203 29922594 PMC5996058

[7] Dong J Li B Lin D Zhou Q Huang D. Advances in targeted therapy and immunotherapy for non-small cell lung cancer based on accurate molecular typing. Front Pharmacol. (2019) 10:230. 10.3389/fphar.2019.00230 30930778 PMC6424010

[8] Gainor JF Dardaei L Yoda S Friboulet L Leshchiner I Katayama R. Molecular mechanisms of resistance to first- and second-generation ALK inhibitors in ALK-rearranged lung cancer. Cancer Discov. (2016) 6:1118–33. 10.1158/2159-8290.CD-16-0596 27432227 PMC5050111

[9] Gandara DR Paul SM Kowanetz M Schleifman E Zou W Li Y. Blood-based tumor mutational burden as a predictor of clinical benefit in non-small-cell lung cancer patients treated with atezolizumab. Nat Med. (2018) 24:1441–8. 10.1038/s41591-018-0134-3 30082870

[10] Gherardi E Birchmeier W Birchmeier C Vande Woude G. Targeting MET in cancer: rationale and progress. Nat Rev Cancer. (2012) 12 89–103. 10.1038/nrc3205 22270953

[11] Goldberg ME Montesion M Young L Suh J Greenbowe J Kennedy M. Multiple configurations of EGFR exon 20 resistance mutations after first- and third-generation EGFR TKI treatment affect treatment options in NSCLC. PLoS ONE. (2018) 13:e0208097. 10.1371/journal.pone.0208097 30481207 PMC6258560

[12] Haanen JB Robert C. Immune checkpoint inhibitors. Prog Tumor Res. (2015) 42:55–66. 10.1159/000437178 26382943

[13] Han B Tjulandin S Hagiwara K Normanno N Wulandari L Laktionov K. EGFR mutation prevalence in Asia-Pacific and Russian patients with advanced NSCLC of adenocarcinoma and non-adenocarcinoma histology: the IGNITE study. Lung Cancer. (2017) 113 37–44. 10.1016/j.lungcan.2017.08.021 29110846

[14] Hellmann MD Ciuleanu TE Pluzanski A Lee JS Otterson GA Audigier-Valette C. Nivolumab plus ipilimumab in lung cancer with a high tumor mutational burden. N Engl J Med. (2018) 378 2093–104. 10.1056/NEJMoa1801946 29658845 PMC7193684

[15] Zakaria N Satar NA Abu Halim NH Ngalim SH Yusoff NM Lin J. Targeting lung cancer stem cells: research and clinical impacts. Front Oncol. (2017) 7:80. 10.3389/fonc.2017.00080 28529925 PMC5418222

[16] Tiran V Lindenmann J Brcic L Heitzer E Stanzer S Tabrizi-Wizsy NG. Primary patient-derived lung adenocarcinoma cell culture challenges the association of cancer stem cells with epithelial-tomesenchymal transition. Sci Rep. (2016) 7:10040. 10.1038/s41598-017-09929-0 28855609 PMC5577216

[17] Borghaei H Paz-Ares L Horn L Spigel DR Steins M Ready NE. Nivolumab versus docetaxel in advanced nonsquamous non-small-cell lung cancer. N Engl J Med. (2015) 373:1627–39. 10.1056/NEJMoa1507643 26412456 PMC5705936

[18] Brahmer J Reckamp KL Baas P Crinò L Eberhardt WE Poddubskaya E. Nivolumab versus docetaxel in advanced squamous-cell non-small-cell lung cancer. N Engl J Med. (2015) 373:123–35. 10.1056/NEJMoa1504627 26028407 PMC4681400

[19] Fehrenbacher L Spira A Ballinger M Kowanetz M Vansteenkiste J Mazieres J Atezolizumab versus docetaxel for patients with previously treated non-small-cell lung cancer (POPLAR): a multicentre, openlabel, phase 2 randomised controlled trial. Lancet. (2016) 387:1837–46. 10.1016/S0140-6736(16)00587-0 26970723

[20] Herbst RS Baas P Kim DW Felip E Pérez-Gracia JL Han JY Pembrolizumab versus docetaxel for previously treated, PDL1-positive, advanced non-small-cell lung cancer (KEYNOTE-010): a randomised controlled trial. Lancet. (2016) 387:1540–50. 10.1016/S0140-6736(15)01281-7 26712084

[21] Rittmeyer A Barlesi F Waterkamp D Park K Ciardiello F von Pawel J Atezolizumab versus docetaxel in patients with previously treated non-small-cell lung cancer (OAK): a phase 3, open-label, multicenter randomised controlled trial. Lancet. (2017) 389:255–65. 10.1016/S0140-6736(16)32517-X 27979383 PMC6886121

[22] Reck M Rodríguez-Abreu D Robinson AG Hui R Csoszi T Fülöp A Pembrolizumab versus chemotherapy for PD-L1-positive nonsmall-cell lung cancer. N Engl J Med. (2016) 375:1823–33. 10.1056/NEJMoa1606774 27718847

[23] Carbone DP Reck M Paz-Ares L Creelan B Horn L Steins M. First-line nivolumab in stage IV or recurrent non-smallcell lung cancer. N Engl J Med. (2017) 376:2415–26. 10.1056/NEJMoa1613493 28636851 PMC6487310

[24] Gettinger S Horn L Jackman D Spigel D Antonia S Hellmann M. Five-year follow-up of nivolumab in previously treated advanced non-small-cell lung cancer: results from the CA209–003 study. J Clin Oncol. (2018) 36:1675–84. 10.1200/JCO.2017.77.0412 29570421

[25] Huang YH Zhu C Kondo Y Anderson AC Gandhi A Russell A. CEACAM1 regulates TIM-3-mediated tolerance and exhaustion. Nature. (2015) 517:386–90. 10.1038/nature13848 25363763 PMC4297519

[26] Ribas A. Adaptive immune resistance: how cancer protects from immune attack. Cancer Discov. (2015) 5:915–9. 10.1158/2159-8290.CD-15-0563 26272491 PMC4560619

[27] Champiat S Dercle L Ammari S Massard C Hollebecque A Postel-Vinay S. Hyperprogressive disease is a new pattern of progression in cancer patients treated by anti-PD-1/PD-L1. Clin Cancer Res. (2017) 23:1920–8. 10.1158/1078-0432.CCR-16-1741 27827313

[28] Ledford H. Promising cancer drugs may speed tumours in some patients. Nature. (2017) 544:13–4. 10.1038/nature.2017.21755 28383004

[29] Hu Y Fu L. Targeting cancer stem cells: a new therapy to cure cancer patients. Am J Cancer Res. (2012) 2:340–56. 22679565 PMC3365812

[30] Morrison R Schleicher SM Sun Y Niermann KJ Kim S Spratt DE. Targeting the mechanisms of resistance to chemotherapy and radiotherapy with the cancer stem cell hypothesis. J Oncol. (2011) 2011:941876. 10.1155/2011/941876 20981352 PMC2958340

[31] Codony-Servat J Rosell R. Cancer stem cells and immunoresistance: clinical implications and solutions. Transl Lung Cancer Res. (2015) 4:689–703. 10.3978/j.issn.2218-6751.2015.12.11 26798578 PMC4700228

[32] Wang P Gao Q Suo Z Munthe E Solberg S Ma L. Identification and characterization of cells with cancer stem cell properties in human primary lung cancer cell lines. PLoS ONE. (2013) 8:e57020. 10.1371/journal.pone.0057020 23469181 PMC3587631

[33] Rivera C Rivera S Loriot Y Vozenin MC Deutsch E. Lung cancer stem cell: new insights on experimental models and preclinical data. J Oncol. (2011) 2011:549181. 10.1155/2011/549181 21209720 PMC3010697

[34] Sullivan JP Minna JD Shay JW. Evidence for self-renewing lung cancer stem cells and their implications in tumor initiation, progression, and targeted therapy. Cancer Metastasis Rev. (2010) 29:61–72. 10.1007/s10555-010-9216-5 20094757 PMC2864581

[35] Lapidot T Sirard C Vormoor J Murdoch B Hoang T Caceres-Cortes J. A cell initiating human acute myeloid leukaemia after transplantation into SCID mice. Nature. (1994) 367:645–8. 10.1038/367645a0 7509044

[36] Bhatia M Wang JC Kapp U Bonnet D Dick JE. Purification of primitive human hematopoietic cells capable of repopulating immune-deficient mice. Proc Natl Acad Sci USA. (1997) 94:5320–5. 10.1073/pnas.94.10.5320 9144235 PMC24676

[37] Dalerba P Dylla SJ Park IK Liu R Wang X Cho RW. Phenotypic characterization of human colorectal cancer stem cells. Proc Natl Acad Sci USA. (2007) 104:10158–63. 10.1073/pnas.0703478104 17548814 PMC1891215

[38] Al-Hajj M Wicha MS Benito-Hernandez A Morrison SJ Clarke MF. Prospective identification of tumorigenic breast cancer cells. Proc Natl Acad Sci USA. (2003) 100:3983–8. 10.1073/pnas.0530291100 12629218 PMC153034

[39] Hermann PC Huber SL Herrler T Aicher A Ellwart JW Guba M. Distinct populations of cancer stem cells determine tumor growth and metastatic activity in human pancreatic cancer. Cell Stem Cell. (2007) 1:313–23. 10.1016/j.stem.2007.06.002 18371365

[40] Singh SK Hawkins C Clarke ID Squire JA Bayani J Hide T Identification of human brain tumor initiating cells. Nature. (2004) 432:396–401. 10.1038/nature03128 15549107

[41] O'Brien CA Pollett A Gallinger S Dick JE A human colon cancer cell capable of initiating tumor growth in immunodeficient mice. Nature. (2007) 445:106–10. 10.1038/nature05372 17122772

[42] Ricci-Vitiani L Lombardi DG Pilozzi E Biffoni M Todaro M Peschle C. Identification and expansion of human colon-cancer-initiating cells. Nature. (2007) 445:111–5. 10.1038/nature05384 17122771

[43] Prince ME Sivanandan R Kaczorowski A Wolf GT Kaplan MJ Dalerba P. Identification of a subpopulation of cells with cancer stem cell properties in head and neck squamous cell carcinoma. Proc Natl Acad Sci USA. (2007) 104:973–8. 10.1073/pnas.0610117104 17210912 PMC1783424

[44] Li C Heidt DG Dalerba P Burant CF Zhang L Adsay V. Identification of pancreatic cancer stem cells. Cancer Res. (2007) 67:1030–7. 10.1158/0008-5472.CAN-06-2030 17283135

[45] Collins AT Berry PA Hyde C Stower MJ Maitland NJ. Prospective identification of tumorigenic prostate cancer stem cells. Cancer Res. (2005) 65:10946–51. 10.1158/0008-5472.CAN-05-2018 16322242

[46] Rodriguez-Torres M Allan AL. Aldehyde dehydrogenase as a marker and functional mediator of metastasis in solid tumors. Clin Exp Metastasis. (2016) 33:97–113. 10.1007/s10585-015-9755-9 26445849 PMC4740561

[47] Zakaria N Yusoff NM Zakaria Z Widera D Yahaya BH Inhibition of NF-κB signaling reduces the stemness characteristics of lung cancer stem cell. Front Oncol. (2018) 8:166 10.3389/fonc.2018.00166 29868483 PMC5966538

[48] Tomita H Tanaka K Tanaka T Hara A. Aldehyde dehydrogenase 1A1 in stem cells and cancer. Oncotarget. (2016) 7: 11018–32. 10.18632/oncotarget.6920 26783961 PMC4905455

[49] Jiang F Qiu Q Khanna A Todd NW Deepak J Xing L. Aldehyde dehydrogenase 1 is a tumor stem cell-associated marker in lung cancer. Mol Cancer Res. (2009) 7:330–8. 10.1158/1541-7786.MCR-08-0393 19276181 PMC4255559

[50] Alamgeer M Ganju V Szczepny A Russell PA Prodanovic Z Kumar B. The prognostic significance of aldehyde dehydrogenase 1A1 (ALDH1A1) and CD133 expression in early stage non-small cell lung cancer. Thorax. (2013) 68:1095–104. 10.1136/thoraxjnl-2012-203021 23878161 PMC3841805

[51] Cortes-Dericks L Froment L Boesch R Schmid RA Karoubi G. Cisplatin-resistant cells in malignant pleural mesothelioma cell lines show ALDH^high^CD44^+^ phenotype and sphere-forming capacity. BMC Cancer. (2014) 14:304. 10.1186/1471-2407-14-304 24884875 PMC4021184

[52] Sullivan JP Spinola M Dodge M Raso MG Behrens C Gao B Aldehyde dehydrogenase activity selects for lung adenocarcinoma stem cells dependent on Notch signalling. Cancer Res. (2010) 70:9937–48. 10.1158/0008-5472.CAN-10-0881 21118965 PMC3058307

[53] Aponte PM Caicedo A. Stemness in cancer: stem cells, cancer stem cells, and their microenvironment. Stem Cells Int. (2017) 2017:5619472. 10.1155/2017/5619472 28473858 PMC5394399

[54] Sodja E Rijavec M Koren A Sadikov A Korošec P Cufer T. The prognostic value of whole blood *SOX2, NANOG* and *OCT4* mRNA expression in advanced small-cell lung cancer. Radiol Oncol. (2016) 50:188–96. 10.1515/raon-2015-0027 27247551 PMC4852967

[55] Kahlert C Bergmann F Beck J Welsch T Mogler C Herpel E. Low expression of aldehyde dehydrogenase 1A1 (ALDH1A1) is a prognostic marker for poor survival in pancreatic cancer. BMC Cancer. (2011) 11:275. 10.1186/1471-2407-11-275 21708005 PMC3135572

[56] Zhou X Wang G Sun Y. A reliable parameter to standardize the scoring of stem cell spheres. PLoS ONE. (2015) 10:e0127348. 10.1371/journal.pone.0127348 25973895 PMC4431815

[57] Zhang DG Jiang AG Lu HY Zhang LX Gao XY. Isolation, cultivation and identification of human lung adenocarcinoma stem cells. Oncol Lett. (2015) 9:47–54. 10.3892/ol.2014.2639 25435932 PMC4246672

[58] Steibel JP Poletto R Coussens PM Rosa GJ. A powerful and flexible linear mixed model framework for the analysis of relative quantification RT-PCR data. Genomics. (2009) 94:146–52. 10.1016/j.ygeno.2009.04.008 19422910

[59] Lin L Jou D Wang Y Ma H Liu T Fuchs J. STAT3 as a potential therapeutic target in ALDH+ and CD44+/CD24+ stem cell-like pancreatic cancer cells. Int J Oncol. (2016) 49:2265–74. 10.3892/ijo.2016.3728 27748818 PMC5118001

[60] Liu A Yu X Liu S. Pluripotency transcription factors and cancer stem cells: small genes make a big difference. Chin J Cancer. (2013) 32:483–7. 10.5732/cjc.012.10282 23419197 PMC3845564

[61] Zhao W Li Y Zhang X. Stemness-related markers in cancer. Cancer Transl Med. (2017) 3:87–95. 10.4103/ctm.ctm_69_16 29276782 PMC5737740

[62] Carney DN Gazdar AF Bunn PA Guccion JG. Demonstration of the stem cell nature of clonogenic tumor cells from lung cancer patients. Stem Cells. (1982) 1:149–64. 6294885

[63] Salvador J Diaz-Cano. Tumor heterogeneity: mechanisms and bases for a reliable application of molecular marker design. Int J Mol Sci. (2012) 13:1951–2011. 10.3390/ijms13021951 22408433 PMC3292002

[64] Dobbin ZC Landen CN. Isolation and characterization of potential cancer stem cells from solid human tumors – potential applications. Curr Protoc Pharmacol. (2013) 63:Unit−14.28. 10.1002/0471141755.ph1428s63 24510756 PMC4041697

[65] Greve B Kelsch R Spaniol K Eich HT Götte M Flow cytometry in cancer stem cell analysis and separation. Cytometry Part A. (2012) 81A: 284–93. 10.1002/cyto.a.22022 22311742

[66] Bielecka ZF Maliszewska-Olejniczak K Safir IJ Szczylik C Czarnecka AM. Three-dimensional cell culture model utilization in cancer stem cell research. Biol Rev. (2017) 92:1505–20. 10.1111/brv.12293 27545872

[67] Broakley KWR Hunn MK Farrand KJ Price KM Grasso C Miller RJ. Side population is not necessary or sufficient for a cancer stem cell phenotype in glioblastoma multiforme. Stem Cells. (2011) 29:452–61. 10.1002/stem.582 21425408

[68] Wang L Zuo X Xie K Wei D. The role of CD44 and cancer stem cells. Methods Mol Biol. (2018) 1692:31–42 10.1007/978-1-4939-7401-6_3 28986884

[69] Park E Park SY Sun PL Jin Y Kim JE Jheon S. Prognostic significance of stem cell-related marker expression and its correlation with histologic subtypes in lung adenocarcinoma. Oncotarget. (2016) 7:42502–12. 10.18632/oncotarget.9894 27285762 PMC5173151

[70] Karachaliou N Rosell R Viteri S. The role of SOX2 in small cell lung cancer, lung adenocarcinoma and squamous cell carcinoma of the lung. Transl Lung Cancer Res. (2013) 2:172–9. 10.3978/j.issn.2218-6751.2013.01.01 25806230 PMC4367598

